# Knowledge, attitudes, and practices of healthcare providers toward gender dysphoria in Saudi Arabia

**DOI:** 10.3389/frhs.2026.1767076

**Published:** 2026-04-10

**Authors:** Waled M. Albalawi, Mohammed A. Alhassan, Muhammad H. Aldossary, Jamal Alothaim, Abdulaziz S. Albalawi, Khalil A. Alghalayini, Majed B. Abaalkhail, Samah H. Alkhawashki

**Affiliations:** 1 King Salman Armed Forces Hospital, Tabuk, Saudi Arabia; 2 Division of Psychiatry, Department of Medical Specialties, College of Medicine, Majmaah University, Al Majmaah, Saudi Arabia; 3 Eradah and Mental Health Complex, Qassim, Saudi Arabia; 4 Department of Psychiatry, King Saud University Medical City, King Saud University, Riyadh, Saudi Arabia; 5 Eradah Mental Health Complex, Riyadh, Saudi Arabia; 6 Department of Psychiatry, College of Medicine, King Saud University, Riyadh, Saudi Arabia

**Keywords:** cultural perceptions, gender dysphoria, healthcare providers, knowledge, attitudes, and practices, practices, medical education, Saudi Arabia, transgender health

## Abstract

**Background:**

Gender dysphoria (GD) involves a marked incongruence between an individual's experienced gender and their sex assigned at birth. Individuals with GD often experience a strong desire to be treated as another gender and frequently face significant health disparities, including limited access to care, higher risks of depression, and suicide. These challenges are often exacerbated by healthcare providers' knowledge gaps and discriminatory attitudes. This study's objective is to assess the knowledge, attitudes, and practices (KAP) of healthcare providers in Saudi Arabia regarding GD and to identify gaps that could inform targeted educational interventions.

**Methods:**

A cross-sectional online survey was conducted with healthcare providers in Saudi Arabia (Nov 2024–Jun 2025). A total of 156 responses were gathered. The questionnaire included domains of knowledge (9 items), attitude (11 items), and practice (4 items) on a 7-point Likert scale. Missing responses, including “I don't know,” were imputed. Descriptive statistics were used to summarize participant responses. Associations among knowledge, attitude, and practice scores were examined using Spearman's rank-order correlation, with additional subgroup analyses conducted to explore variations by demographic characteristics.

**Results:**

Participants demonstrated moderate to strong knowledge and generally positive attitudes toward individuals with GD, particularly regarding pronoun use, mental health assessment, and awareness of health disparities. However, practical engagement was limited, with only 19% reporting direct clinical experience and a few having received GD-related training. Scores varied by age, training level, and specialty, as respondents aged 24–39 years showed the strongest knowledge and attitude scores, and practice scores were highest among those aged 40–49 years, while senior registrars demonstrated the highest overall KAP scores, and psychiatry showed the strongest overall specialty profile.

**Conclusion:**

Healthcare providers demonstrate foundational knowledge and supportive attitudes but limited practical experience in managing GD. Targeted training and structured clinical exposure are recommended to improve culturally competent and inclusive care.

## Introduction

According to the Diagnostic and Statistical Manual of Mental Disorders, Fifth Edition, Text Revision (DSM-5-TR, 2022), gender dysphoria (GD) is characterized by a significant mismatch between an individual's experienced or expressed gender identity and their assigned gender at birth (1). GD is distinguished from gender variance which is an expression of deviation from societal gender norms. While gender identity is one's understanding of being male, female or nonbinary, on an individual basis, sexual orientation is related to attraction. GD, as a result of mismatches between gender identity and assigned sex, has significant effects on mental health and therefore on the need for an affirming care (1, 2). The prevalence of GD has increased notably in recent years, and the condition may lead to clinically significant distress and impairment in social, occupational, or other important areas of functioning (3–5).

The prevalence of GD has typically been estimated through two main approaches: in adults, by monitoring the number of individuals seeking care from gender identity clinics or related health services; and in younger populations, through large-scale studies that often rely on parental reports of gender-related experiences and behaviors or on self-report measures (6, 7). Estimating the prevalence of GD from only those seeking medical or legal assistance may be an underestimation of the true extent of GD, as many do not access medical care because of stigma, shame, financial limitations, or fear of relationship disruption. Individuals struggling with societal discrimination and internal struggles often suffer from increased rates of anxiety, depression and suicidal ideation, with the coming out process adding to their feelings of loneliness and rejection (2, 7, 8).

Recent evidence indicates that gender diversity is increasingly recognized across different populations. A systematic review and meta-analysis reported a steady rise in the prevalence of GD over the past five decades, reaching 4.6 per 100,000 adults, 6.8 per 100,000 among those assigned male at birth and 2.6 per 100,000 among those assigned female at birth (9). Similarly, a population-based study in Taiwan found that the prevalence of GD in 2019 had nearly doubled compared with that observed in 2010 (10). A national survey conducted in the United Kingdom further reported that approximately 1% of adults identified as gender diverse or transgender (11). In the Netherlands, the prevalence of clinically defined GD was estimated at 0.6% among adults assigned male at birth and 0.2% among those assigned female at birth (7).

Individuals with GD face substantial health disparities, including barriers to healthcare access, higher rates of suicide attempts, and an increased prevalence of depression (12). These challenges are primarily driven by discrimination and stigma within healthcare systems, compounded by insufficient provider knowledge of GD-specific health needs (13). Furthermore, individuals with GD often experience a strong desire to be treated as another gender, or as an alternative gender different from their sex assigned at birth, or to be free from their primary and secondary sex characteristics, which can lead to a range of negative consequences. Therefore, assessing healthcare providers' knowledge is crucial to ensure competent, inclusive, and sensitive care for individuals with GD (10).

Many studies report discrimination against gender-diverse individuals in healthcare; for example, the 2015 U.S. Transgender Survey found that one-third of respondents experienced at least one negative healthcare event in the past year due to their gender identity. In a mixed-methods systematic review involving predominantly American, Canadian, European, and South African-based participants, Cruciani et al. reported that sexual and reproductive health practitioners often demonstrated uncertainty about appropriate practices, as well as discomfort and implicit biases, which limited their ability to provide affirming care for transgender, gender-diverse, and non-binary patients (14). Another study of nurse practitioners in the northern United States found that, despite their good intentions, a lack of experience and education in transgender healthcare limited their ability to fulfill their role effectively (15). A cross-sectional survey of 631 Italian general practitioners assessing their knowledge of transgender and gender-diverse health found that younger practitioners were more likely than older colleagues to answer related questions accurately. Additionally, 66% of participants agreed that transgender individuals often feel afraid or anxious about accessing healthcare services (16).

Despite growing recognition of GD as a significant healthcare concern, research exploring healthcare providers' knowledge, attitudes, and practices (KAP) in the Middle East remains limited. Available evidence is sparse; for example, the Lebanese Medical Association has initiated efforts to develop context-appropriate clinical guidance, while studies from Iran have evaluated the impact of online educational interventions aimed at improving healthcare professionals' knowledge of GD (17). In Saudi Arabia, a national mental health committee recently characterized GD as a psychological illness without an organic or hormonal basis, emphasizing psychological treatment and discouraging hormone therapy or gender-affirming surgeries, which are considered medically inappropriate and potentially harmful according to that committee's guidance (18). In many Middle Eastern countries, there are no specific legal protections or established healthcare pathways for transgender individuals, and in the absence of locally developed protocols, medical professionals often rely on Western clinical guidelines.

This gap limits understanding of how well-prepared professionals are to deliver competent, sensitive care to individuals with GD. To address this issue, the primary objective of this study is to assess healthcare workers' current knowledge and attitudes in Saudi Arabia, with particular focus on their understanding of the condition, available treatment options, and potential biases influencing patient care. The secondary objective is to examine existing practices for managing patients with GD and identify educational deficiencies and training needs to inform the development of targeted programs that foster more inclusive, patient-centred healthcare practices.

## Methodology

### Study design and setting

This study employed a cross-sectional questionnaire-based design. The survey was distributed online via social media platforms (e.g., WhatsApp) and through the Saudi Commission for Health Specialties' email distribution list to maximize reach among healthcare professionals in Saudi Arabia. The study was conducted between November 2024 and June 2025.

### Target population

The target population included healthcare providers currently practicing across Saudi Arabia, who could potentially encounter patients experiencing GD in the course of their general medical practice. The survey did not specifically target physicians providing specialized GD-related care, but rather health care providers representing a broad range of medical disciplines who may encounter such cases incidentally. Individuals who were unwilling to participate were excluded from the study. In addition, individuals who were unable to communicate effectively in either English or Arabic were also excluded.

### Sample size and sampling technique

The sample size was calculated using the formula:

$$N=(Z^{2}\times P\times (1-P))/D^{2}$$

where *Z* = 1.96, *p* = 50%, and *D* = 5%. The minimum required sample size was estimated to be 384.

### Questionnaire development

The survey instrument was adapted from multiple open-access questionnaires previously used for academic and research purposes, with minimal modifications to align with the local cultural context. The final questionnaire included three constructs: Knowledge (9 items), Attitude (11 items), and Practice (4 items). All items were assessed using a seven-point Likert scale with response options ranging from 1 = Strongly Disagree, 2 = Disagree, 3 = Somewhat Disagree, 4 = Neither Agree nor Disagree, 5 = Somewhat Agree, 6 = Agree, and 7 = Strongly Agree. Higher scores indicated greater knowledge, more positive attitudes, and more appropriate or supportive practices regarding GD. An additional “I don't know” option was included; such responses were treated as missing and imputed along with other missing values. Reverse coding was applied to negatively worded items. For item A11 (“Gender dysphoria is a condition that can be treated”), the term “treated” was intended to reflect the provision of appropriate professional care, including psychological support and, where clinically indicated, gender-affirming medical management. The questionnaire did not explicitly define the term within the survey instrument; therefore, responses were based on participants' individual interpretations.

### Study procedures and data collection

Data were collected via an online survey platform on Google Forms, consisting of multiple-choice and Likert-scale items. The questionnaire underwent pilot testing among approximately 15 healthcare professionals to ensure clarity, reliability, and validity. In addition, to assess the internal consistency of the study constructs following data imputation, Cronbach's alpha coefficients were calculated for both the raw and imputed datasets. Feedback from this pilot was incorporated to refine the final version. The finalized questionnaire was then disseminated online via email, social media platforms including LinkedIn, and WhatsApp, with a brief introduction explaining the study's purpose, significance, and estimated completion time. Informed consent was obtained electronically before participation as participants were required to read the informed consent statement before beginning the survey. Anonymity and confidentiality were assured, and no personally identifiable information was collected. The platform ensured secure data storage, protected anonymity, and allowed direct export of responses to statistical software (RStudio, version 4.5.0) for analysis.

The survey initially gathered responses from 298 participants. However, due to substantial attrition and incomplete responses, only respondents who completed at least 80% of the survey items were retained for analysis to ensure the integrity of results. The final sample size was 156. For the remaining missing data, median imputation stratified by sex was applied.

### Confidentiality and ethical considerations

Ethical approval was obtained from the King Saud University Medical City Research Ethics Committee. A cover page presented the project title, objectives, and intended use of findings. Consent was implied by survey completion, with participants informed that their responses would remain confidential and that they could withdraw at any time. A study contact email was provided for inquiries or withdrawal requests. Data were securely stored and accessible only to the research team. Furthermore, Ethical approval was obtained from the Saudi Commission for Health Specialties to disseminate the survey to all registered healthcare providers.

### Statistical analysis

The survey comprised three constructs: Knowledge (9 items), Attitude (11 items), and Practice (4 items). Each item was rated on a seven-point Likert scale, with an additional option for “I don't know.” Responses marked as “I don't know” were treated as missing data and imputed alongside other missing values, as including them as a valid scale point would distort the integrity of the seven-point structure.

Missing responses were addressed using a straightforward imputation strategy. Specifically, missing values were replaced with the modal response within each sex stratum, thereby preserving sex-specific response tendencies while reducing potential bias from nonresponse. For the Knowledge domain, missing values were imputed; however, the response “I don't know” was coded as 0 to ensure it carried no weight in the summation of individual scores, while still indicating lack of knowledge.

Computation of KAP scores involved summing item responses across all questions within each construct. To enhance comparability and interpretability, these summed scores were standardized by dividing them by the maximum possible score for the respective construct and then expressed on a normalized 0–100 scale. This transformation facilitated direct comparison across constructs and between raw and imputed datasets, while maintaining fidelity to the original scoring framework.

Given the ordinal nature of the data, Spearman's rank-order correlation was used to assess relationships among the KAP scores. Correlations were also examined within stratified subgroups. Correlations with *p*-values less than 0.05 indicate statistically significant differences in median scores.

All analyses were conducted in RStudio using the “stats” and “rstatix” packages.

## Results

### Descriptive analysis

#### Demographic and classification variables

Table 1 shows the distribution of respondents across demographic variables and clinical classifications. A total of 156 respondents participated in the study. The sample was predominantly female (55%), with males comprising 45%. The majority were aged 24–29 years (56%), followed by those aged 30–39 (31%). Participants aged 40 and above accounted for a smaller proportion (12%). Regional representation was highest from the Central region (46%), with the Western (24%), Eastern (15%), Southern (8%), and Northern (8%) regions contributing the remainder.

**Table 1:** Frequency distribution of respondents by demographic variables (*n* = 156).

Variable	Count	Percentage
Sex		
Female	86	55%
Male	70	45%
Age group		
24–29	88	56%
30–39	49	31%
40–49	10	6%
50–59	4	3%
60+	5	3%
Region		
Central	71	46%
Western	37	24%
Eastern	23	15%
Southern	13	8%
Northern	12	8%

Table 2 presents the frequency distribution of respondents by clinical classification. The most represented clinical classifications were consultants (21%) and psychologists (21%), followed by social workers (15%) and third-year residents and higher (16%). In terms of specialty, psychiatry comprised the largest group (34%), followed by psychology (21%) and social work (15%).

**Table 2:** Frequency distribution of respondents by clinical classifications (*n* = 156).

Variable	Count	Percentage
Classification		
Consultant	33	21%
Psychologist	32	21%
Resident 3+	25	16%
Social worker	24	15%
Resident 1/2	21	13%
Senior registrar	11	7%
Fellow	6	4%
Medical student	3	2%
Other	1	1%
Specialty and subspecialty		
Psychiatry	53	34%
Psychology	32	21%
Social work	24	15%
Family medicine	12	8%
Emergency	8	5%
Internal medicine	6	4%
Endocrinology	1	1%
General	3	2%
Genetics	1	1%
Pulmonology	1	1%
Ob-Gyne	4	3%
Neurology	3	2%
Urology	3	2%
Public health	2	1%
Pediatrics	2	1%
General surgery	2	1%
Nursing	1	1%
Radiology	1	1%
Physiatry	1	1%
Intern	1	1%
Pediatric neurology	1	1%

#### Overall KAP

**Figure 1:**
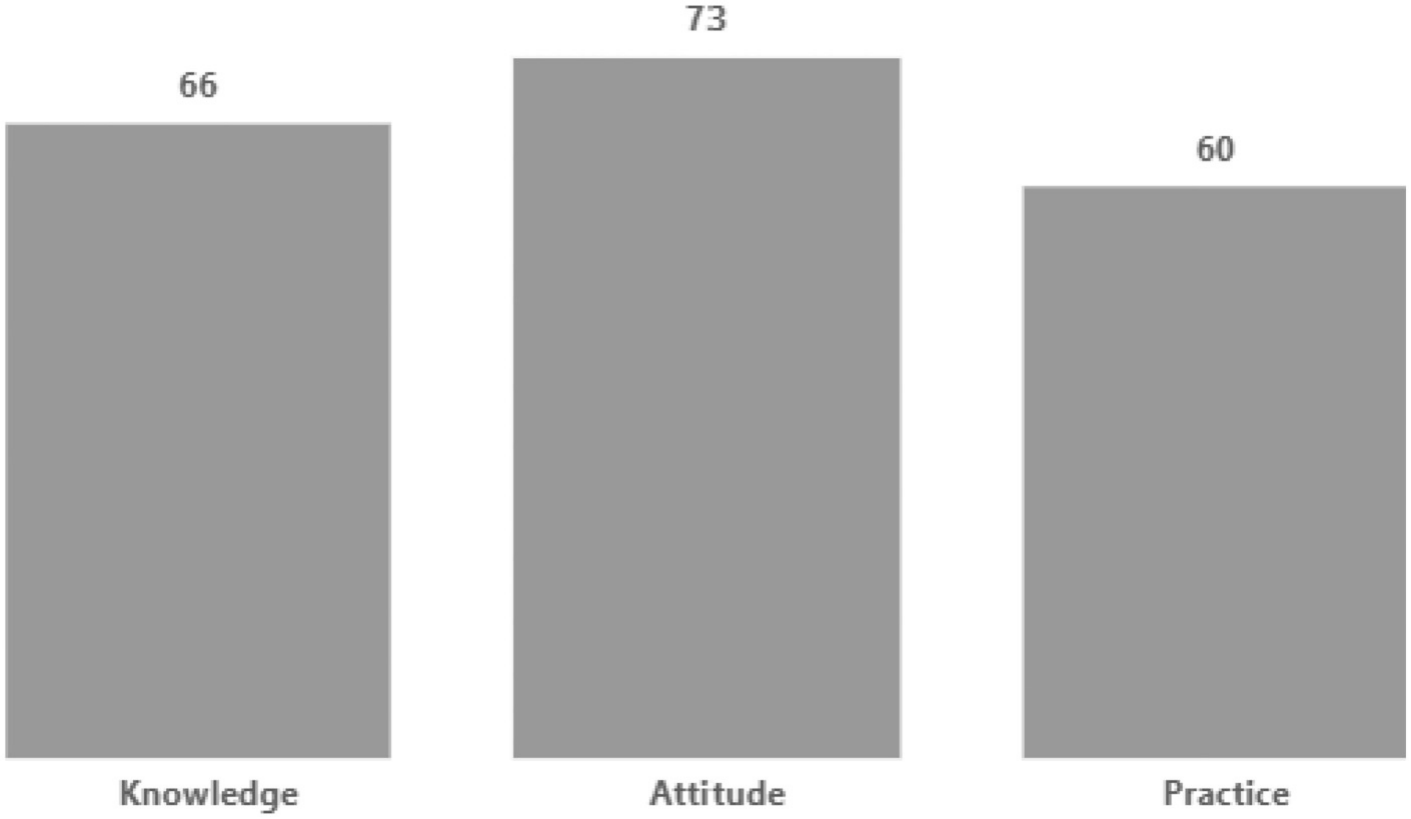
Summary of the overall KAP score of the respondents (*n* = 156).

Figure 1 shows the distribution of individual overall scores for each construct. Results indicated that respondents demonstrated the strongest performance in the Attitude domain, with a mean score of 73, while Knowledge (66) and Practice (60) scores were comparatively lower.

#### Knowledge domain

Table 3 presents the percentage distribution of responses to knowledge-related items rated on a 7-point Likert scale, ranging from Strongly Disagree to Strongly Agree, with Neither Agree nor Disagree as the midpoint. The two response categories with the highest percentages for each item are highlighted. Item-level analysis of the Knowledge domain indicated variable familiarity with transgender healthcare concepts. High agreement on items K1, K2, K3, K7, and K9 demonstrated strong awareness of appropriate pronoun use and recognition of health disparities, with over 50% of respondents endorsing these statements. In contrast, items K4, K6, and K8 showed greater proportions of uncertainty and disagreement, highlighting a limited understanding of clinical protocols and treatment criteria. Responses to reverse-worded items (K2, K7, K8) suggested adequate comprehension, particularly for K2, where 52% correctly differentiated gender identity from sexual orientation. K8, however, remained an area of persistent misconception, with high disagreement rates.

**Table 3:** Item-level response distribution for knowledge items (%).

Knowledge items	Strongly agree	Agree	Some what agree	Neither agree nor disagree	Some what disagree	Disagree	Strongly disagree
K1. I am knowledgeable about the healthcare needs of individuals with gender dysphoria.	27	28	20	9	3	8	6
K2^a^. Gender identity and sexual orientation are two different names for two different concepts.	37	15	9	8	12	9	11
K3. An individual must be diagnosed with gender dysphoria by a qualified mental health professional to receive hormone replacement therapy.	47	19	11	6	3	6	8
K4. I have a clear understanding of the fundamental principles of hormone therapy and sex reassignment surgery.	10	13	11	26	6	15	19
K5. An individual experiencing gender dysphoria should be addressed using their preferred pronouns rather than those referring to their sex assigned at birth.	19	17	18	15	4	6	20
K6. I understand the difference between puberty blockers and cross-hormone therapy, as well as when it is appropriate to suggest or prescribe either.	10	10	12	33	4	14	17
K7^a^. To be considered gender dysphoric, an individual is not required to undergo at least one gender-affirming surgery.	33	24	13	11	10	4	6
K8^a^. At this time, gender dysphoria treatments are considered suitable topics within conventional medicine.	9	13	4	11	29	19	14
K9. I am aware of the health disparities and inequities faced by patients who identify as gender dysphoric in our society.	31	38	9	13	3	1	4

^a^ Items K2, K7, and K8 are reverse-worded to ensure conceptual balance for the overall score and correlations.

#### Attitude domain

Table 4 presents the percentage distribution of responses to attitude-related items rated on a 7-point Likert scale, ranging from Strongly Disagree to Strongly Agree, with Neither Agree nor Disagree as the midpoint. The two response categories with the highest percentages for each item are highlighted. Item-level analysis of the Attitude domain demonstrated predominantly supportive views toward GD care. High agreement across items A1–A7 reflected respondents' comfort, sense of responsibility, and empathy in providing care. Items A8 and A9 indicated moderate comfort in managing male-to-female and female-to-male individuals, though some residual discomfort was reported. Reverse-worded items A3 and A4 were largely disagreed with, suggesting minimal stigma and general willingness to engage with GD patients. Responses to A10 showed mixed opinions regarding the perceived increase in GD incidence in Saudi Arabia, while A11 demonstrated broad agreement that GD is a treatable condition, however, with no emphasis on the type of treatment provided.

**Table 4:** Item-level response distribution for attitude items (%).

Attitude items	Strongly agree	Agree	Some what agree	Neither agree nor disagree	Some what disagree	Disagree	Strongly disagree
A1. All physicians have a responsibility to provide care for patients experiencing gender dysphoria.	50	19	10	11	3	2	6
A2. I would feel comfortable if my professional peers were aware that I treat patients with gender dysphoria.	37	31	8	13	0	2	9
A3^a^. I am not concerned that if my other patients find out I am treating patients with gender dysphoria, they may no longer seek my care.	39	29	6	8	3	4	10
A4^a^. I might accept to provide care for patients with gender dysphoria due to the potential complexities involved.	31	22	11	10	8	10	8
A5. I am comfortable discussing gender dysphoria with my seniors/colleagues.	29	26	19	8	3	6	8
A6. I understand the fears of intolerance and discrimination that individuals with gender dysphoria might experience when seeking healthcare.	49	24	15	6	2	1	3
A7. As an individual or healthcare provider, I would feel comfortable interacting with an individual with gender dysphoria.	27	22	19	12	4	8	8
A8^a^. I do not expect to feel discomfort when providing care for male-to-female transgender (MTF) individuals.	17	15	4	19	13	15	17
A9^a^. I do not expect to feel discomfort when providing care for female-to-male transgender (FTM) individuals.	19	17	5	21	6	15	17
A10. I believe that the incidence of gender dysphoria has increased in Saudi Arabia over the last 10 years.	19	37	18	12	6	4	4
A11. I believe that gender dysphoria is a condition that can be treated.	23	46	13	6	1	7	4

^a^ Items A3, A4, A8, and A9 are reverse-worded to ensure conceptual balance for the overall score and correlations.

#### Practice domain

Table 5 presents the percentage distribution of responses to practice-related items rated on a 7-point Likert scale, ranging from Strongly Disagree to Strongly Agree, with Neither Agree nor Disagree as the midpoint. The two response categories with the highest percentages for each item are highlighted. Responses to the practice items revealed significant gaps in both experiential exposure and curricular integration. For P1, only a minority of participants (19%) reported direct experience in treating patients with GD, while 29% strongly disagreed. In contrast, P2 indicated relatively high self-reported confidence in taking medical histories, with 61% scoring strongly agree or agree, suggesting some preparedness despite minimal hands-on experience. Items P3 and P4 highlighted curricular shortcomings: responses were widely dispersed, and only about one-third of participants affirmed that GD healthcare was included or adequately covered in their training. Notably, 26% strongly disagreed with P4, suggesting perceived deficiencies in educational preparation.

**Table 5:** Item-level response distribution for practice items (%).

Practice items	Strongly agree	Agree	Some what agree	Neither agree nor disagree	Some what disagree	Disagree	Strongly disagree
P1. I had the opportunity to participate in the treatment of a patient with gender dysphoria.	19	21	15	7	2	7	29
P2. I feel confident and prepared to take a medical history from a patient experiencing gender dysphoria.	26	35	9	11	7	7	5
P3. Gender dysphoria healthcare is an essential component of my medical training, and it is included in the medical curriculum.	18	17	12	17	2	11	24
P4. I feel that my medical education prepared me to provide competent and compassionate care for patients experiencing gender dysphoria.	15	9	8	15	3	24	26

### KAP scores by demographic variables

**Figure 2:**
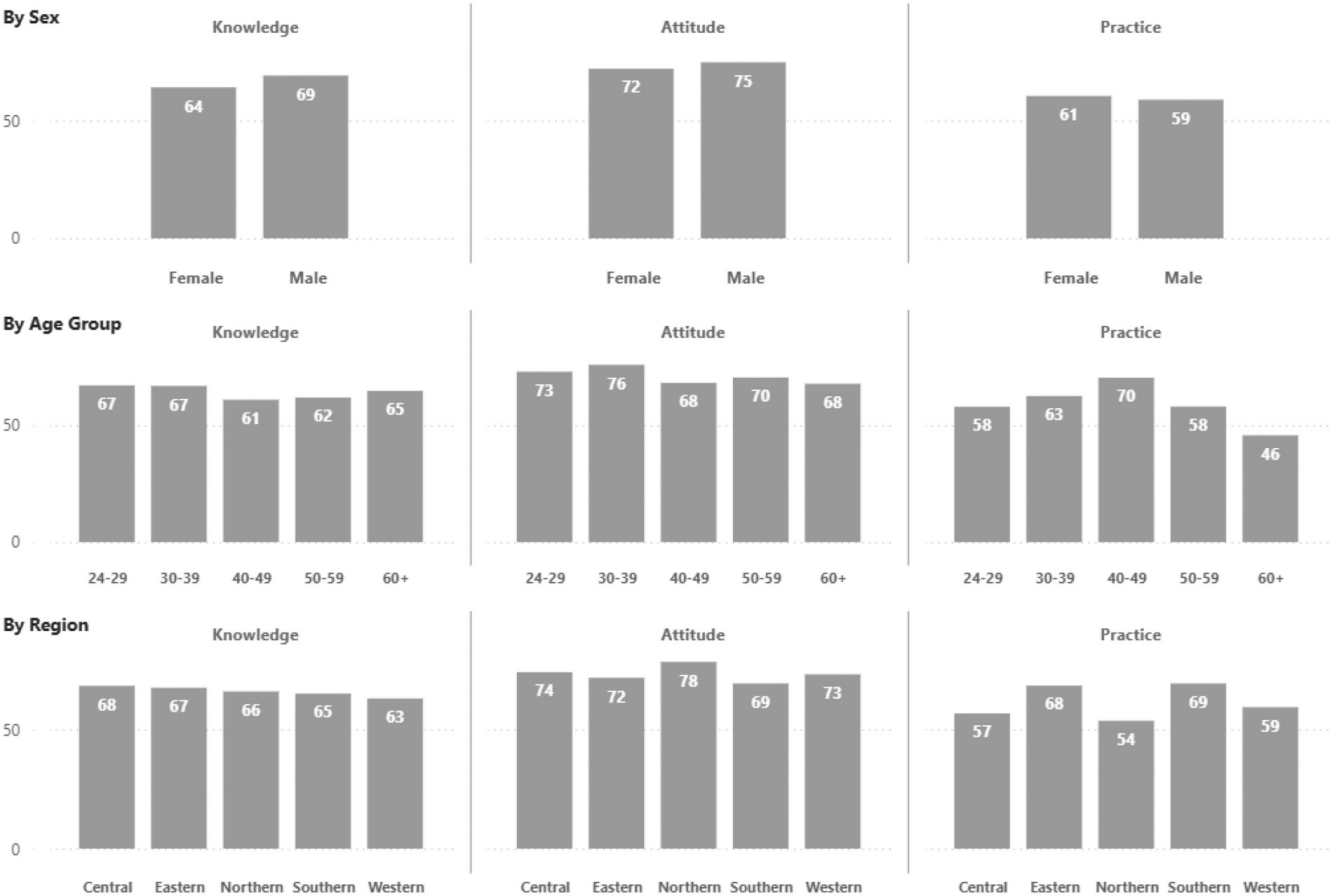
Summary of average KAP scores of the respondents by demographic variables.

Analysis of KAP scores by demographic variables revealed modest variations across sex, age, and region (Figure 2). Male respondents demonstrated slightly higher than females across all domains, with the largest difference observed in knowledge (69 vs. 64). Attitude scores were also higher among males (75 vs. 72), while practice showed only a modest gap (59 vs. 61). Age-related trends revealed that respondents aged 24–29 and 30–39 achieved the highest knowledge scores (67 each), while attitudes peaked in the 30–39 group (76). Practice was strongest among those aged 40–49 (70), but declined sharply in the 60+ group (46). Regional comparisons showed that attitudes were strongest in the Northern region (78) and Central region (74), while practice was highest in the Southern (69) and Eastern (68) regions. Knowledge was most pronounced in the Central (68) and Eastern (67) regions (Figure 2).

### KAP scores by classification and specialty

**Figure 3:**
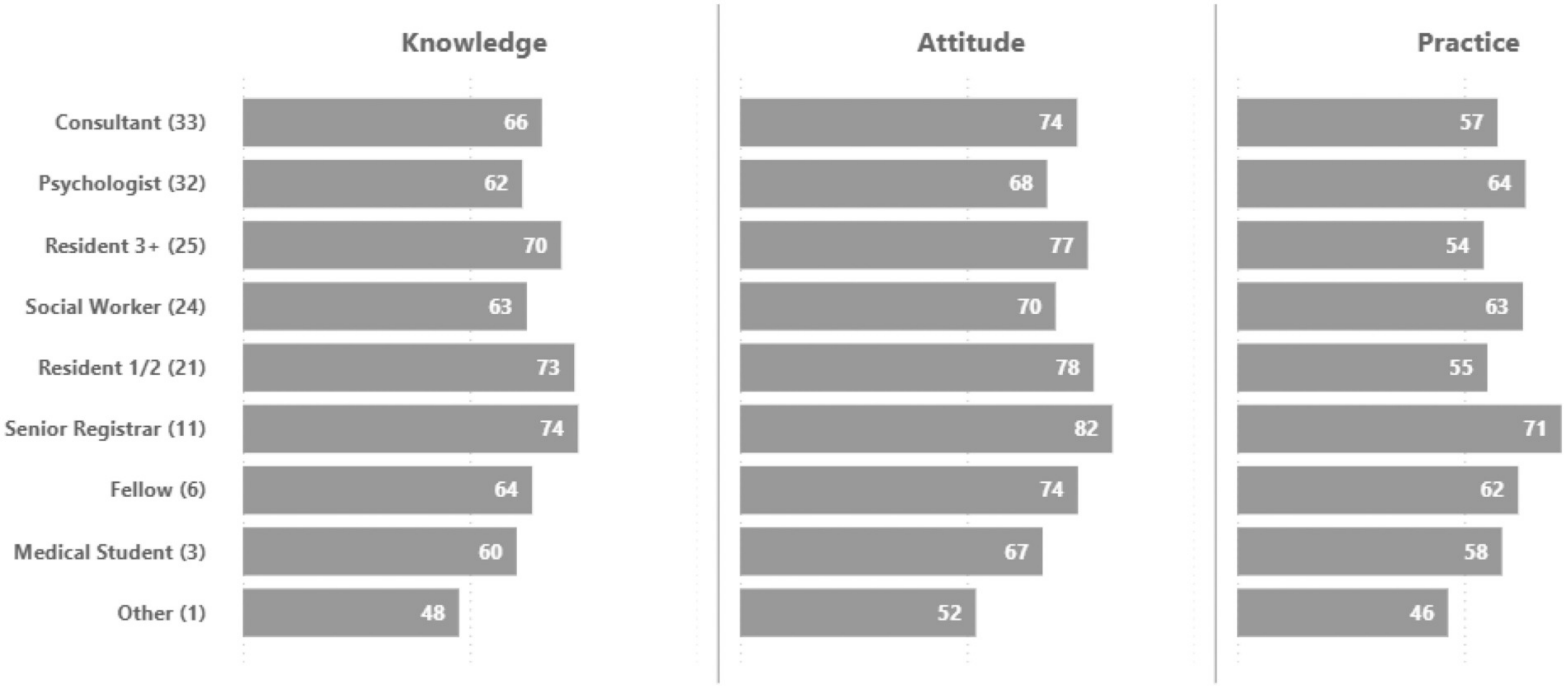
Summary of average KAP scores of the respondents by classification (*n*).

Across professional roles, attitudes toward GD treatment were consistently higher than knowledge and practice (Figure 3). Senior Registrars (*n* = 11) demonstrated the most favorable attitudes (82) alongside strong knowledge (74) and practice (71). Junior residents (Resident 1/2) (*n* = 21) also performed well, reporting high knowledge (73) and attitudes (78), though practice was comparatively lower (55). Social Workers (*n* = 24) and Psychologists (*n* = 32) showed balanced profiles, with solid knowledge (63 and 62) and practice (63 and 64). Consultants (*n* = 33) and Fellows (*n* = 6) reported moderate scores across domains, while Medical Students (*n* = 3) and the “Other” category (*n* = 1) recorded the lowest values, reflecting limited exposure or training.

**Figure 4:**
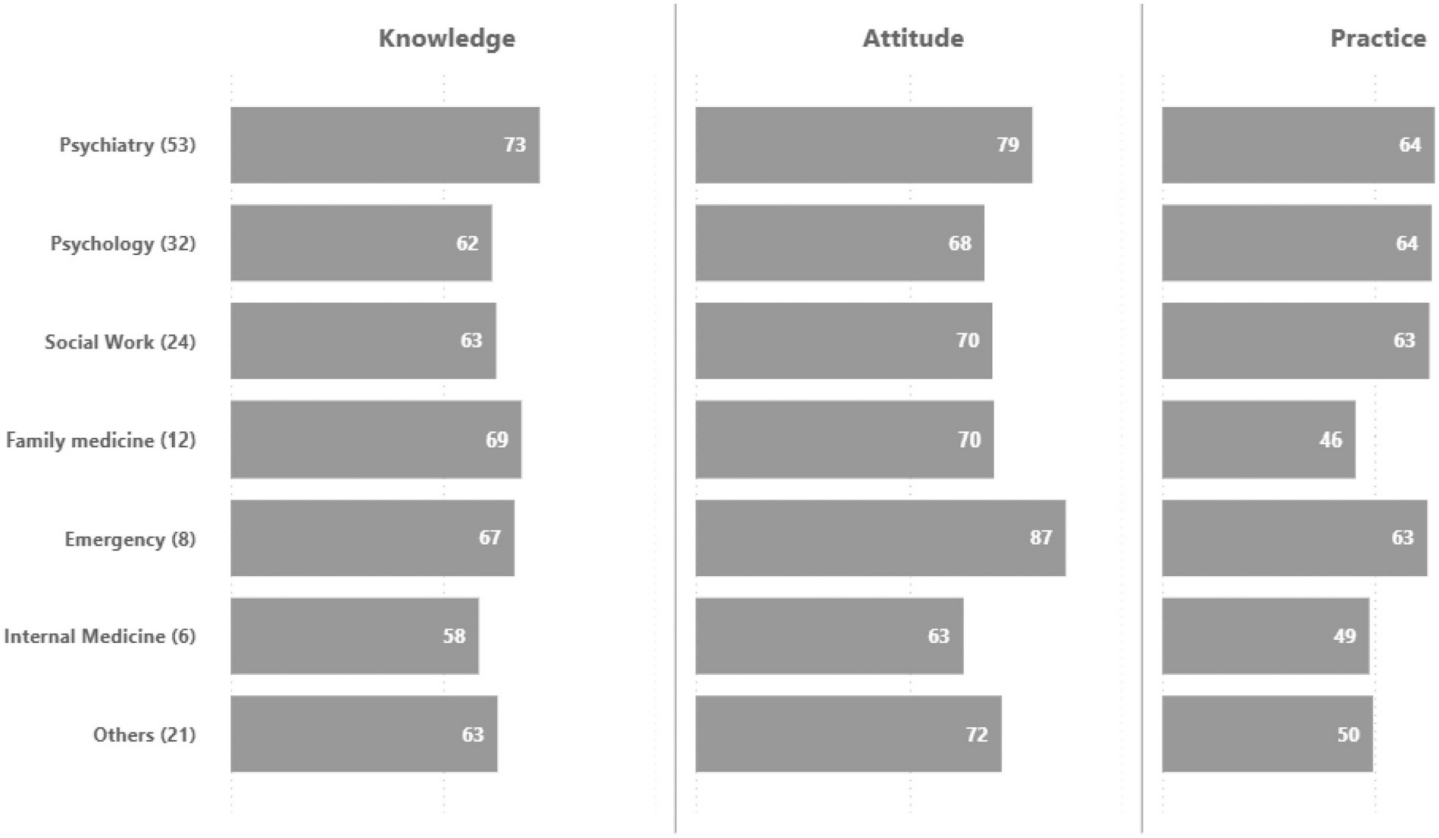
Summary of average KAP scores of the respondents by specialty (*n*).

Analysis of KAP scores by specialty demonstrated notable variability across disciplines (Figure 4). Among professional groups, Psychiatry (*n* = 53), Psychology (*n* = 32), and Social Work (*n* = 24) demonstrated relatively high and balanced scores across knowledge (62–73), attitude (68–79), and practice (63–64), reflecting stronger familiarity and engagement with GD treatment. Emergency Medicine (*n* = 8) stood out with the highest attitude score (87), though practice (63) was moderate. In contrast, Family Medicine (*n* = 12) and Internal Medicine (*n* = 6) reported lower scores across domains, particularly practice (46 and 49).

### Reliability measures

As shown in Table 6, the reliability estimates remained relatively stable across conditions. For the Knowledge construct, alpha decreased slightly from 0.58 (raw) to 0.54 (imputed), suggesting marginal sensitivity to missing data handling. The Attitude construct demonstrated strong reliability in both datasets (0.77 raw; 0.76 imputed), indicating robustness of measurement. For Practice, alpha values were moderately consistent (0.67 raw; 0.64 imputed).

**Table 6:** Reliability coefficients (Cronbach's alpha) for raw and imputed data (*n* = 156).

Construct	Raw data	Imputed data
Knowledge	0.58	0.54
Attitude	0.77	0.76
Practice	0.67	0.64

Overall, the *post-hoc* reliability analysis indicates that imputation did not substantially alter the internal consistency of the scales. While minor reductions were observed, the pattern of coefficients suggests that the constructs retained acceptable psychometric properties, particularly for Attitude and Practice. These findings support the defensibility of the imputation procedure and reinforce the stability of the measurement model across analytic conditions.

### Correlation analysis

#### Overall

Spearman correlation coefficients were computed to examine the associations among KAP constructs under both raw and imputed datasets. In the raw data, Knowledge and Attitude demonstrated a strong positive correlation (*ρ* = 0.64, *p* < 0.0001), which remained significant though slightly attenuated after imputation (*ρ* = 0.56, *p* < 0.0001). The relationship between Attitude and Practice was moderate in the raw dataset (*ρ* = 0.43, *p* < 0.0001), but weakened considerably following imputation (*ρ* = 0.26, *p* = 0.0012). Similarly, the correlation between Knowledge and Practice decreased from moderate (*ρ* = 0.47, *p* < 0.0001) to weaker levels (*ρ* = 0.30, *p* = 0.0003) after imputation (Table 7).

**Table 7:** Summary of Spearman correlation coefficient (*ρ*_s_) and significance levels (*n* = 156).

Constructs	Raw data		Imputed data	
	*ρ*_s_	*p*-value	*ρ*_s_	*p*-value
K vs. A	0.64	<0.0001*	0.56	<0.0001*
A vs. P	0.43	<0.0001*	0.26	0.0012*
K vs. P	0.47	<0.0001*	0.30	0.0003*

* Significant at α = 0.05.

#### By sex

Across sex groups, females demonstrated consistently stronger and more significant correlations among the three variables compared to males (Table 8). While males showed a moderate and significant association between K and A, their correlations with P were weak and non-significant. In contrast, females exhibited robust correlations across all variable pairs, suggesting that inter-variable relationships may be more pronounced in women.

**Table 8:** Summary of spearman correlation coefficient (*ρ*_s_) and significance levels by demographic variables.

Group	*n*	K vs. A		A vs. P		K vs. P	
		*ρ*_s_	*p*-value	*ρ*_s_	*p*-value	*ρ*_s_	*p*-value
Sex							
Male	70	0.45	0.0003*	0.17	0.2977	0.09	0.4385
Females	86	0.63	<0.0001*	0.33	0.0021*	0.47	<0.0001*
Age group							
24–29	88	0.59	<0.0001*	0.15	0.1693	0.27	0.0197*
30–39	49	0.36	0.0368*	0.20	0.1670	0.31	0.0626
40–49	10	0.79	0.0130*	0.82	0.0120*	0.55	0.1010
50–59	4	0.98	0.0458*	0.94	0.1176	0.90	0.1176
60+	5	0.97	0.0176*	0.52	0.7480	0.36	0.7480
Region							
Central	71	0.53	<0.0001*	0.36	0.0039*	0.30	0.0114*
Western	37	0.71	<0.0001*	0.33	0.0430*	0.44	0.0119*
Eastern	23	0.45	0.0964	−0.08	0.7192	0.27	0.4086
Southern	13	0.63	0.0654	0.14	0.6518	0.30	0.6518
Northern	12	0.43	0.4979	0.42	0.4979	0.09	0.7872

Groups with *n* < 10 are disregarded in the analysis.

* Significant at *α* = 0.05.

#### By age group

Age group analyses revealed a general pattern of increasing correlation strength with age, though small sample sizes in older cohorts limit firm conclusions. Among younger adults (24–29 years), K–A correlations were strong and highly significant, with a modest but significant K–P association. The 30–39 group showed only a significant K–A relationship, while those aged 40–49 displayed very strong correlations across all pairs, albeit based on a small sample. The oldest groups (50–59 and 60+) exhibited extremely high correlation coefficients, but statistical significance was inconsistent due to limited sample sizes, warranting cautious interpretation (Table 8).

#### By region

Regional comparisons highlighted notable heterogeneity. The Central and Western regions showed consistent and significant correlations across most variable pairs, with particularly strong K–A associations. By contrast, the Eastern, Southern, and Northern regions demonstrated weaker and largely non-significant correlations, likely reflecting smaller sample sizes and regional variability. These findings suggest that contextual or cultural factors may influence the strength of associations, with Central and Western regions standing out as areas of more robust inter-variable relationships (Table 8).

#### By classification

Consultants and psychologists showed the strongest and most consistent correlations across all variable pairs, with moderate to high coefficients that were statistically significant. In contrast, residents, social workers, and other classifications exhibited weaker or non-significant associations, with small sample sizes further limiting interpretability (Table 9).

**Table 9:** Summary of spearman correlation coefficient (*ρ*_s_) and significance levels by classification.

Classification	*n*	K vs. A		A vs. P		K vs. P	
		*ρ*_s_	*p*-value	*ρ*_s_	*p*-value	*ρ*_s_	*p*-value
Consultant	33	0.62	0.0003*	0.53	0.0028*	0.50	0.0032*
Psychologist	32	0.66	0.0001*	0.40	0.0249*	0.53	0.0035*
Resident 3+	25	0.34	0.2861	0.13	0.7947	0.18	0.7947
Social worker	24	0.37	0.1523	−0.13	0.5305	0.41	0.1410
Resident 1/2	21	0.49	0.0749	0.16	0.9731	−0.04	0.9731
Senior registrar	11	−0.03	1.0000	0.09	1.0000	0.62	0.1211
Fellow	6	0.53	0.8336	−0.39	0.8862	−0.16	0.8862
Medical student	3	0.97	0.4329	0.89	0.5900	0.77	0.5900

Groups with *n* < 10 are disregarded in the analysis.

* Significant at *α* = 0.05.

#### By specialty

Psychology showed the strongest and most consistent correlations across all variable pairs, with significant moderate to high associations. Family medicine and internal medicine also demonstrated strong K–A relationships, though small sample sizes limit generalizability. Psychiatry revealed a moderate but significant K–A correlation, while social work and emergency medicine showed weaker, non-significant associations. Other specialties such as Ob-Gyne, neurology, and urology produced extreme coefficients, but their very small sample sizes make these results unreliable (Table 10).

**Table 10:** Summary of spearman correlation coefficient (*ρ*_s_) and significance levels by specialty.

Specialty	*n*	K vs. A		A vs. P		K vs. P	
		*ρ*_s_	*p*-value	*ρ*_s_	*p*-value	*ρ*_s_	*p*-value
Psychiatry	53	0.36	0.0253*	0.24	0.1653	0.24	0.1653
Psychology	32	0.66	0.0001*	0.40	0.0249*	0.53	0.0035*
Social work	24	0.37	0.1523	−0.13	0.5305	0.41	0.1410
Family medicine	12	0.76	0.0121*	0.15	1.0000	−0.06	1.0000
Emergency	8	0.32	1.0000	0.28	1.0000	0.38	1.0000
Internal medicine	6	0.91	0.0348*	0.51	0.2971	0.70	0.2411
Ob-Gyne	4	−0.87	0.3972	0.75	0.4917	−0.39	0.6088
Neurology	3	0.99	0.3212	−0.68	1.0000	−0.54	1.0000
Urology	3	−1.00	0.1461	−0.11	1.0000	0.04	1.0000

Groups with *n* < 10 are disregarded in the analysis.

* Significant at *α* = 0.05.

## Discussion

According to the Committee for Professional and Ethical Practices in Mental Health, affiliated with the National Center for Mental Health Promotion in Saudi Arabia, GD is considered a psychiatric condition rather than a biological or hormonal one (18). Individuals with this condition have fully developed reproductive organs consistent with their genetic sex (46, XY in males; 46, XX in females), distinguishing it from intersex conditions that may require earlier medical intervention. A key feature of the disorder is non-acceptance of one's assigned gender at birth, often accompanied by a strong psychological desire to alter external or internal physical characteristics (18).

This study contributes to understanding their KAP regarding GD and identifies several key patterns across demographic and professional variables. Translating this knowledge into practice emphasizes the importance of integrating culturally and ethically appropriate guidelines into clinical care.

### Knowledge and conceptual understanding

In this study, the majority of participants (60%) demonstrated a solid grasp of core GD concepts, such as appropriate pronoun use, diagnostic procedures, and awareness of gender-related health disparities. This may be attributed to the gradual integration of gender diversity discourse within clinical and educational settings in Saudi Arabia, consistent with recent literature showing increased familiarity with health terminology among healthcare professionals. For example, a study among Italian general practitioners found that the most respondents (79.1%) correctly answered questions about the sexual orientation of gender-diverse individuals (16). However, their depth of understanding, particularly regarding clinical protocols and treatment criteria, was uneven. Marconi et al. demonstrated that knowledge gaps were especially evident in preventive care, with fewer than half of respondents correctly identifying the need for breast (40.1%) and prostate (36%) cancer screenings (16). This highlights the importance of implementing targeted educational programs to address these specific knowledge gaps (19). A survey of 810 healthcare providers across four European countries found that 63.0% (*n* = 455) preferred gender-diverse healthcare training to be included in their mandatory professional development (20).

In this study, the comparative analysis revealed that male participants scored slightly higher than female participants in the knowledge domain (Figure 2). This may be attributed to younger male generations being culturally aware and sensitive to diversity issues, including gender identity (21).

### Attitudes toward gender dysphoria care

Overall, attitudes toward GD care were highly supportive, with most agreeing that all physicians share responsibility for caring for patients with GD and expressing comfort in discussing such cases with peers or patients themselves. The majority ≈75% expressed willingness to treat GD patients, consistent with emerging regional evidence suggesting shifting professional attitudes toward gender-diverse populations. These findings align with studies from Western contexts that show healthcare providers increasingly endorse affirming perspectives toward gender-diverse individuals. Paradiso et al. reported that nurse practitioners in the U.S. demonstrate compassion, acceptance, and a strong commitment to providing respectful care for transgender patients (15).

Nevertheless, moderate discomfort persisted in items related to providing care to male-to-female and female-to-male patients. This residual hesitation may stem from cultural stigma, limited clinical exposure, or the influence of religious beliefs and societal norms on provider attitudes (22). Bhatt et al. also reported that preventive care and necessary screenings are frequently missed in these patients, due to patient discomfort and reluctance to seek care (23). Persistent discomfort among healthcare providers when managing male-to-female or female-to-male patients may reflect insufficient training, limited clinical exposure, and uncertainty regarding appropriate communication and care practices. Studies have shown that many family physicians feel underprepared to address gender-specific health needs, particularly those related to transition-related care or psychosocial support (24). In gynecological and primary care settings, providers frequently report low confidence in managing female-to-male or male-to-female patients, suggesting the urgent need for standardized clinical guidelines, targeted education, and experiential learning opportunities to enhance provider competence (25).

### Practice and clinical engagement

Despite favorable knowledge and attitudes, practice scores remained the lowest across domains. Only a small proportion of respondents (19%) had direct experience managing patients with GD, and many indicated that their medical education had not adequately prepared them for such cases. Nevertheless, self-reported confidence in basic clinical tasks, such as taking medical histories, was relatively high. This discrepancy may reflect reliance on general clinical competencies rather than specific training in gender-affirming care. Approximately half of the respondents disagreed that their medical education had sufficiently prepared them to provide competent and compassionate care for individuals with GD, highlighting persistent deficiencies in curricular coverage and practical training. Without structured guidance, even well-intentioned providers may inadvertently deliver inadequate or insensitive care. Accordingly, enhanced training in cultural competency and adherence to established standards of medical care for GD patients are strongly recommended to promote equitable, informed, and sensitive healthcare delivery (26).

### Demographic and professional variations

Gender, age, and regional differences revealed essential nuances. Males exhibited slightly higher knowledge and attitude scores, potentially reflecting differing educational or social experiences with GD-related topics. Younger professionals demonstrated greater knowledge and more positive attitudes, likely owing to recent academic exposure and greater digital literacy (22); however, their practice scores were lower, suggesting that while awareness is increasing among younger generations, practical implementation still requires structured clinical experience. Conversely, middle-aged professionals exhibited better practice outcomes, likely due to prolonged professional and clinical experience (22). Regional variation, notably higher knowledge and practice scores in the Eastern and Southern regions, may be linked to greater healthcare diversity or exposure to international medical training programs in those areas.

Psychiatry and Emergency Medicine professionals consistently outperformed others across domains, while Internal Medicine practitioners showed the lowest scores. The high performance among psychiatrists is expected, given their frequent encounters with GD-related psychological and diagnostic aspects, as well as their personal exposure to patients, which strongly influences their knowledge and practice (22). Importantly, incremental improvements in knowledge and practice among psychiatry residents across training levels suggest that hands-on exposure during residency effectively enhances competence. Conversely, Family Medicine practitioners, despite high knowledge and attitudes, reported low practice, suggesting a persistent knowledge and practice gap in primary care, a setting critical for early GD recognition and referral.

### Correlation patterns and mediating role of attitude

A moderate positive correlation between knowledge and attitude indicated that greater awareness was associated with more favorable attitudes toward GD. This association was powerful among female respondents, suggesting that improved understanding may more effectively translate into supportive attitudes within this group. In contrast, the relationship was weaker but still significant among males, emphasizing the need for gender-sensitive educational strategies that foster both factual knowledge and empathetic engagement.

A positive association was also observed between attitude and practice, implying that favorable attitudes can facilitate greater clinical engagement and competence in GD care. Among younger respondents, significant positive correlations emerged between knowledge and attitude, suggesting that early-career professionals may be more receptive to translating awareness into action when provided with appropriate guidance and training. This is likely due to the benefits of recent formal education and practical on-the-job exposure (22). Regionally, participants from the Central and Western regions demonstrated the strongest inter-construct associations across all pairs, indicating a more cohesive integration of knowledge, perception, and behavior in GD-related care within that context.

Profession-specific differences further highlighted variability in the relationships among KAP domains. Significant positive correlations across all variable pairs were observed among psychologists and consultants, indicating a closer alignment between knowledge, attitudes, and practice in these groups. Among specialties, psychology showed the strongest overall associations, particularly between knowledge and attitude. This pattern may reflect differences in training background and professional exposure to the psychosocial dimensions of GD-related care.

### Limitations and future directions

This study has several limitations. First, the reliance on self-reported data may introduce response and social desirability bias, particularly given the socially sensitive nature of GD in Saudi Arabia. Second, although a target sample size was calculated, the use of a non-probability convenience sampling strategy limits the representativeness of the findings, and the calculated number should be interpreted as a target rather than a probability-based estimate. The calculated sample size was 384. However, the final analytic sample was 156 due to a predefined 80% completion threshold, resulting in attrition from incomplete responses. This attrition may not have been random. It is possible that providers with lower KAP were more likely to discontinue the survey. If so, the results may be skewed towards reflecting higher KAP scores than are truly representative. The smaller final sample size compared to the calculated target may have reduced the statistical power of the analyses and further limited the generalizability of the findings. Additionally, the sample was disproportionately composed of mental health related professionals, which may further limit the generalizability of the findings across different specialties and regions. Accordingly, the results should be interpreted as exploratory rather than nationally representative.

It is important to acknowledge that the wording of item A11 (“a condition that can be treated”) may have been subject to varying interpretations. While the intended meaning referred to appropriate clinical management and supportive care, some participants may have interpreted “treatment” as conversion to biological sex or as reflecting a pathologizing perspective. This potential ambiguity could have influenced responses and should be considered when interpreting findings within the attitude domain.

Furthermore, the internal consistency of the Knowledge construct was relatively low (Cronbach's alpha ranging from 0.54 to 0.58), which may reflect heterogeneity among the knowledge items and should be considered when interpreting results related to this domain.

Future research should aim for larger, more representative samples, employ longitudinal designs to examine causal pathways, and consider qualitative approaches to gain deeper insights into the barriers and facilitators of healthcare providers' engagement with GD care.

## Conclusion

Healthcare providers in Saudi Arabia demonstrated moderate to strong knowledge and positive attitudes toward GD. However, gaps in clinical practice and limited hands-on experience highlight deficiencies in training and curricular coverage. Knowledge strongly correlated with attitudes, suggesting that targeted educational interventions could enhance both understanding and support for gender-diverse patients. To ensure culturally competent, inclusive, and affirming care, healthcare systems should implement structured training programs, increase clinical exposure, and address barriers to the practical application of gender-affirming care.

## Data Availability

The raw data supporting the conclusions of this article will be made available by the authors, without undue reservation.
